# Reasons for late presentation for antenatal care, healthcare providers’ perspective

**DOI:** 10.1186/s12913-019-4855-x

**Published:** 2019-12-30

**Authors:** Nelly Jinga, Constance Mongwenyana, Aneesa Moolla, Given Malete, Dorina Onoya

**Affiliations:** Department of Internal Medicine, Health Economics and Epidemiology Research Office, School of Clinical Medicine, Faculty of Health Sciences, University of the Witwatersrand, Johannesburg, South Africa

**Keywords:** Antenatal care, Healthcare providers, Late presentation, Midwife, Pregnancy

## Abstract

**Background:**

Antenatal care (ANC) provides healthcare services to pregnant women in an attempt to ensure, the best possible pregnancy outcome for women and their babies. Healthcare providers’ understanding of their patient’s behaviour and reasons for engagement in care and their response to this insight can influence patient-provider interactions and patient demand for ANC early in pregnancy. We examined the insight of healthcare providers into women’s reasons for starting ANC later than the South African National Department of Health’s recommended 20 weeks gestation. We also looked at the impact of late ANC presentation on overall healthcare providers’ work experiences and their response in their interactions with patients.

**Methods:**

In-depth interviews were conducted with 10 healthcare providers at Maternal Obstetrics Units (MOU) and Primary Healthcare Centres (PHC) in Gauteng, South Africa. Healthcare providers were selected with the assistance of the facility managers. Data analysis was conducted using the qualitative analysis software NVivo 11, using a thematic approach of pinpointing, examining, and recording patterns within the data.

**Results:**

Healthcare providers were aware of patients need for secrecy in the early stages of pregnancy because of fears of miscarriage and women’s preference for traditional care. Women with prior pregnancies presumed to know about stages of pregnancy and neglected to initiate ANC early. Barriers to early ANC initiation also include, women’s need to balance income generating activities; travel cost to the clinic and refusal of care for coming after the daily patient limit has been reached. Healthcare providers encounter negative attitudes from un-booked patients. This has a reciprocal effect whereby this experience impacts on whether healthcare providers will react with empathy or frustration.

**Conclusions:**

Timing of ANC is influenced by the complex decisions women make during pregnancy, starting from accepting the pregnancy itself to acknowledging the need for ANC. To positively influence this decision making for the benefit of early ANC, barriers such as lack of knowledge should be addressed prior to pregnancy through awareness programmes. The relationship between healthcare providers and women should be emphasized when training healthcare providers and considered as an important factor that can affect the timing of ANC.

## Background

Antenatal care (ANC) provides healthcare services to the pregnant women in an attempt to ensure, by antenatal preparation, the best possible pregnancy outcome for women and their babies. The care includes screening for pregnancy problems, assessment of pregnancy risk, provision of information to pregnant women and support for women to make pregnancy and birth a positive life experience. ANC also provides routine preventive care offered to pregnant women to aid in the development and delivery of a healthy child [1, 2]. Early presentation for ANC is widely acknowledged to contribute towards improved pregnancy outcomes such as fullterm delivery and normal birth weight [3]. ANC plays a vital role in the early detection and treatment of HIV infection in pregnant women [4]. However, in sub-Saharan Africa, late initiation of ANC is common, though variable by country, region, culture and population [5]. More than 60% of women attend their first ANC visit after 20 weeks gestation [6–11] and some (6.0%) go into labour without having attended any ANC visits [7, 12].

The South Africa Department of Health guidelines recommend that pregnant women seek ANC services as soon as they suspect a pregnancy or as early as the first missed menstrual period. All pregnant women are encouraged to attend eight ANC visits in order to reduce the risk of adverse obstetric outcomes [1]. In South Africa, between 2015 and 2016, 94% of pregnant women attending public health facilities had at least one ANC visit [13]. More recently (2018), three quarters of women were observed to have made at least four ANC visits, 47% had an ANC visit in the first trimester, 32% first received ANC during the fourth or fifth month of pregnancy, 2% delayed care until the eighth month and about 6% were un-booked at Midwife Obstetric Units (MOUs) [14].

Patient-specific factors such as having a history of a miscarriage, obstetric complications, still-birth and nulliparity have been associated with early ANC attendance [15, 16]. However, low-socioeconomic background, lack of partner support, work commitments and not planning a pregnancy are known to promote late ANC presentation in Sub-Saharan Africa [6, 15, 17]. Furthermore, limited access to affordable healthcare services is an important contributor to late ANC presentation in African countries [18]. Even in South Africa, where patients are no more than a ten minute walking distance from a Primary Health Care Centre (PHC), long waiting periods, overcrowding and limited health personnel continue to challenge the provision of quality health services [19, 20]. Ebonwu et al. (2018) found that late ANC presentation in rural South Africa was associated with being married, employed, less than 20 years of age, having an unplanned pregnancy and being pregnant for the first time [18]. The study mentioned above found that being pregnant for the first time is associated with late ANC presentation which contradicts other authors [15, 16] who found nulliparity to be associated with early ANC attendance. In the South African context where a significant number of people believe in witch doctors and being bewitched, fear of being bewitched has been reported as one of the factors associated with late presentation for ANC. Haddad et al. (2016) explored the barriers to early prenatal care in South Africa and the results showed that women presented late because they contemplated abortion, feared the ANC HIV test outcome, jealousy and bewitchment [21].

In South Africa, nurses at Primary Health Care (PHC) centers including Maternal Obstetric Units (MOU), play an important role in antenatal, delivery and postpartum care. Healthcare providers’ negative behaviors and attitudes are often cited as central deterrents of early ANC attendance [22]. However, very little research has probed into healthcare providers’ understanding, and insight into their patients’ care-seeking behavior or the contribution of this insight to their own motivation and commitment to providing quality care. These perspectives are important to outline as healthcare providers perception of their patients’ motivation for seeking care, including the way patients engage with health systems, informs providers’ experience of the provider-patient interaction, quality of service provided and ultimately patients’ care seeking behavior [23].

A study conducted in South Africa demonstrated that the patient-provider relationship can have a far-reaching effect on the women’s ability to fully engage in the prevention of mother to child transmission (PMTCT) steps, starting with early presentation for ANC [24]. A qualitative study from Tanzania found that patient-provider interactions affected patient knowledge, general wellbeing, and ultimately access to and retention in PMTCT services [25]. Although some studies have explored the impact of the provider-patient relationships on patient behaviours; there is limited information on how these interactions shape healthcare workers’ understanding of patients’ health care seeking motivators and their care-giving experience and responses, particularly in the provision of PMTCT-related care. We aimed to examine the insights of healthcare providers into women’s reasons for starting ANC than the South African National Department of Health’s recommended 20 weeks gestation. We also looked at the impact of late presentation for ANC on overall antenatal service provision (healthcare providers’ experiences and response).

## Methods

### Study setting and design

We conducted a qualitative cross-sectional study in Gauteng province, South Africa. Participants were recruited from Community Health Centres (CHC) which often includes a Primary Health Care Centre (PHC) and Midwife Obstetric Unit (MOU). Staff at CHCs include enrolled nurses, registered nurses, nursing assistants, community health workers and a visiting medical officer. Antenatal appointments occur mainly at a PHC, however MOUs are required to provide HIV counselling and testing services, as well as antiretroviral treatment (ART) initiation when these did not occur before labour. In addition, MOUs also provide early postpartum care. Healthcare providers were selected using purposive sampling with the assistance of the facility managers based on their involvement in antenatal care (ANC) and with the aim of having representation of the various healthcare providers. With guidance from the facility managers all potential eligible healthcare providers were approached in-person to assess their interest in participating in the study. Those interested were then asked for their availability and an appointment was scheduled to obtain written informed consent and conduct the semi-structured interview. Healthcare providers were eligible for inclusion if they were either seeing patients for antenatal care, counselling for HIV testing during pregnancy or involved in the delivery of babies. Prior to conducting interviews, informed consent was obtained individually from all study participants.

### Data collection

Interviews were conducted by trained researchers. The semi-structured interview guide had questions probing on healthcare providers’ knowledge and perspectives on pregnant women’s reasons for late presentation for their first antenatal visit, their experience of ANC provision and how they responded to health care challenges that they faced. The interview guide is summarized in Table 1. The interviews were 30–60 min long and conducted in English. All interviews were audio-recorded with the permission of the participants. Interviewing was continued until no new themes emerged and data saturation was reached which resulted in a total of ten participants being interviewed.

**Table 1:** An overview of the semi-structured interview guide

Theme	Primary question asked
Healthcare providers’ perspective on ANC presentation by pregnant women	Why do you think women present late for the first ANC visit? Why do you think they are lost during antenatal care?
Challenges healthcare providers face in implementing ANC	What are the challenges you or your colleagues face in implementing ANC and PMTCT program in relation to clients that you see?
Healthcare providers reaction to challenges they face in implementing ANC	What are the challenges you or your colleagues face in implementing ANC and PMTCT in relation with your capacity?

### Data analysis

The audio recordings were transcribed verbatim by a trained transcriber. All transcripts were quality controlled by the study team who replayed the audios while reading the transcripts to maintain accuracy. Data analysis was conducted using the qualitative analysis software NVivo 11. Data were analysed using a thematic approach of pinpointing, examining, and recording patterns within the data. Initially deductive, pre-assigned codes were used based on a conceptual framework built around providers’ perspectives on reasons for ANC late presentation. This was followed by inductive code development to deepen the data analysis. Two transcripts were completely coded by three researchers and compared for inter-coder reliability and the codebook for analysing the remaining transcripts was thereafter revised and finalized. To overcome bias, transcripts were coded by 3 researchers, two of the researchers CM and NJ had conducted the interviews and one researcher AM had not conducted the interviews and DO was also involved in checking and verifying the interpretations of the data.

### Ethical considerations

This study was approved by the University of the Witwatersrand’s Human Research Ethics Committee (HREC) (Clearance certificate No. M151041). Permission to conduct the study was obtained from Tshwane Health District, City of Johannesburg Health District and Ekurhuleni Health District. Written informed participant consent was obtained for each individual in-depth interview.

## Results

### Participants characteristics

All ten healthcare providers interviewed were female. Three were registered nurses, four were nurse practitioners and three were midwives. Out of the three midwives, one was a facility manager. They had work experience ranging from 3 years to 19 years.

### Overview of perceived reasons for late presentation of ANC

**Fig. 1:**
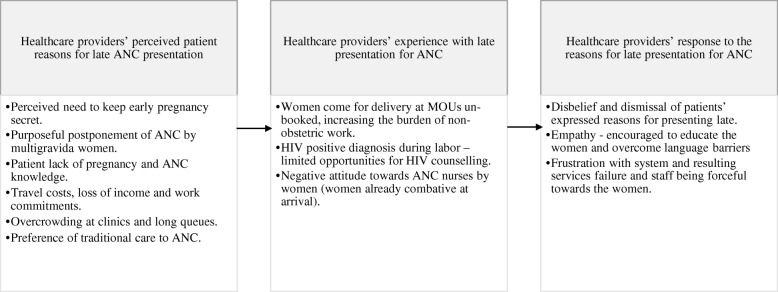
Healthcare providers’ perceived patient reasons for late ANC presentation, their experience of ANC services provision, responses to patient reasons

Healthcare providers highlighted a variety of perceived patient reasons for late presentation for ANC services at individual patient, clinic and community/cultural level. Figure 1 shows the interplay between, perceived patient reasons for presenting late, healthcare providers’ responses to these reasons and how it influences their attitude towards women.

### Healthcare providers’ perceived patient reasons for late presentation for ANC

#### Perceived need to keep early pregnancy secret

Healthcare providers reported that most women were reluctant to disclose a pregnancy before 4 months gestation and start ANC due to cultural beliefs about the vulnerability of the pregnancy in the early stages, and the need to safeguard the pregnancy by keeping it a secret from friends and family. Attendance of ANC services will require disclosure of the pregnancy and ANC is therefore postponed until disclosure is inevitable.

*“Sometimes they are not sure about the pregnancy, they want to make sure, and at least if they are four months pregnant then they can say now they are pregnant. … We cannot run away from our culture. Two months’ pregnancy they will say “wow, two months? isale metsi (that is still water), just keep it to yourself.” All this make people not to think about going for ANC before the twenty weeks.”* (Midwife, MOU)

In some case this avoidance of pregnancy disclosure is due to the fear of stigma when the woman is unmarried/ underage, older or the pregnancy is unplanned, especially soon after a previous pregnancy. In some cases, women struggle to accept and welcome the unplanned pregnancy.

*“The other reason why will they come late is that a person is having a baby of less than a year old. So that shame of people knowing that I have another baby. It’s the one that makes them late.”* (Nurse practitioner, MOU)

#### Purposeful postponement of ANC by multigravida women

Healthcare providers perceived that women with previous pregnancies, especially older women, did not value ANC services in early pregnancy and purposefully postponed ANC attendance. This was attributed to women’s perceived experience and knowledge gained in prior pregnancies. There was an impression that some pregnant women prefer home deliveries as opposed to coming into the clinic.

*“Some they think they have experience especially if they have been pregnant for more than 3 times …”* (Registered nurse, MOU)

*“Some of them because it is the second baby, they tell themselves that they know they can deliver themselves at home.”* (Midwife, MOU)

Negative ANC experiences during previous pregnancies were thought to be important deterrents to early ANC attendance among older women.

*“The young ones, most of the time they book. The problem is the old ones they tell themselves that they know, and the nurses have got attitude…”* (Midwife, MOU)

#### Women’s lack of pregnancy and ANC knowledge

Healthcare providers reported that a general lack of knowledge about early signs of pregnancy was an important contributor to late presentation for ANC. Women do not know the optimal time to start ANC and the benefits of receiving ANC throughout pregnancy, with many attending only to secure a booking for obstetric services.

*“Some are not sure about their last date of menstruation. Some didn’t know that they were pregnant because they will say ‘I was seeing my menstrual cycle’* (Registered nurse, MOU)

*“… Others know but they will say I will go when I am about to deliver but I think most of the people don’t know when to report for ANC.”* (Midwife, MOU)

#### Travel costs, loss of income and work commitments

Healthcare providers were aware of the challenge posed by travel costs in starting ANC and remaining adherent to the subsequent visit schedule.

*“Yes, transport money, sometimes they default, and they say, “sister on this day I didn’t have money to come, I was waiting for my husband to get paid”* (Nurse practitioner, MOU)

However, providers also felt that transportation could no longer be a major deterrent as recent developments have shortened the travel distance to primary health care centres in South Africa.

*‘It’s (transport money) not a problem, its close-by, they can walk. It’s reachable. It’s just that they show no interest these women. Because sometimes you find that through her ANC, she went to the clinic only to book so that she gets the card and come here to deliver. When you check the ANC card, she went there once. The results are still there; she never went back to get the results. Treatment is still behind”.* (Midwife, MOU)

However, healthcare providers acknowledged that employed women may struggle to come early for ANC because of work commitments. According to healthcare providers, employed women from the communities that they serve may not have maternity benefits or paid leave to attend. As a result, unless physically unwell, attending ANC is of lower priority compared to other, more pressing, responsibilities.

*“...Some will tell you I am working, and I don’t have time and we are not allowed time to go to the clinic…”* (Midwife, MOU)

#### Overcrowding at clinics and long queues

Healthcare workers admitted that women endured long waiting times and were sometimes turned away because of limited staff to see to all patients. In some cases, clinics imposed daily quotas and turned away women who came after the quota was reached

*“The other one would tell you that they have been turned away from the clinic because there were too many patients. Other clinics, they work from 7h00 to 16h00. Probably there are too many patients and they cannot see all of them in a day, some patients must be turned away* (Registered nurse, MOU)

*“...Some will tell you that I was going to the clinic and I was put off by the line because if you are new, they are taking a certain number (maybe 20 per day). So, when I come late it will be late for me...”* (Midwife, MOU)

A midwife at an MOU when asked about the challenges that healthcare providers face due to late presentation of ANC and how the government can support, had this to say;

*“Increase staffing. Also counsellors should be there to assist the sisters (nurses and midwives). It is like the sisters are being traumatized somehow. Because you need to see twenty patients on the line, five of them are new and among them maybe four are positive.* (Midwife, MOU)

#### Preference of traditional care to ANC

Healthcare providers reported that some women seem to prefer traditional/religious healers who may misinterpret pregnancy symptoms for other sicknesses. This impacts the timing of first ANC visit, since these women believe that they are not pregnant.

*“Religious reasons, some will tell you that they went to church and they told me there is a snake moving in my belly, yeah those kind of stories… and after 8 months when we tell them that they are pregnant, they will be surprised.”* (Registered nurse, MOU)

### Healthcare providers’ experience with late presentation for ANC

Presenting late for ANC doesn’t only impact on the mother and baby but it also impacts the health system, healthcare providers and the care that they are able to provide to women. Obstetric Units are impacted in particular, as they deal with the compounded care needs in the later stages of pregnancy and childbirth.

#### Women come for delivery at MOUs un-booked

Some women were reported to not attend ANC at all during their pregnancy. When these women went into labour, the nurses had to do ANC–specific tests and administer preventive interventions, including HIV counselling and testing procedures. When un-booked and undiagnosed women present for delivery in late afternoons or at night when no lay counsellors are available at MOUs, midwives take on the counsellor role as well. HIV diagnostic processes are time consuming and put additional pressure on midwives who are likely to rush through HIV counselling and consenting for HIV treatment and prevention interventions during labour. MOU nurses also reported that they sometimes must postpone HIV testing and ART initiation until after delivery, increasing the risk of vertical transmission of HIV to exposed infants. In some cases, women even refuse the offer to test when they are in labour.

*“We do have ladies that come here un-booked and we don’t know their status. So, if somebody comes here right in labour and she is about to give birth we don’t know her status. It’s a bit challenging because, you know, some do come here while the baby is about to come out and then by then you haven’t tested them because they are to get their medicine. After delivery then we start testing and give the treatment whereas treatment is supposed to be given immediately and the tablets were supposed to be given before delivery, others don’t want to be tested. They don’t want to know their HIV status at all. Such people we don’t know what to do with them.”* (Midwife, MOU)

*“The emotional part of women being in labour and having labour pains. We do HIV tests and it comes out positive. There is no time to calm the woman, there is nothing such as that. The woman is in pain, sometimes women don’t understand what you are talking about because they are focused on the pain. At that moment you still have to try hard and explain and make sure that the they understand what is going on, so that the woman should not just get treatment every three hours not knowing what exactly is going on.” (Midwife, MOU)*

#### Pregnant women’s negative attitude towards healthcare providers

Late presentation for ANC also presents a challenge to midwives because when women start ANC late, they would come prepared to defend themselves against any possible negative staff attitude. They come with an anticipation that the healthcare providers will verbally abuse them for not attending ANC, and in some cases, healthcare providers also do respond in such an expected negative manner.

*“There is attitude, from both of us. Sometime the client will come in having this negative attitude or having heard from other people that there is this and she just came in, even if she was not mistreated, she comes in prepared, fighting. By the time you ask her questions, she is not nice. Because of some of the things that are being done by her, then the staff will start to be negative towards her.”* (Midwife, MOU)

### Healthcare providers’ response to late presentation for ANC

In their day to day interactions with pregnant women, healthcare providers responded in different ways to the work pressure resulting from patients presenting late for ANC. Healthcare providers reacted with both empathy and frustration to women presenting late for ANC. Healthcare providers found it difficult to believe some of the reasons women gave for presenting late and they also reported frustration with system failures such as shortage of staff and the resulting long queues. However, sometimes they realised that women acted out of lack of knowledge and nurses were motivated to educate them.

#### Disbelief

As women presented late for ANC, especially when presenting un-booked at the MOU (delivery site), they explained to healthcare workers why they presented late. Healthcare providers refused to believe some of the explanations that women gave which had the potential of breaking down respectful and empathic provider-patient interaction.

*“Yeah you can find somebody came in un-booked, and she has got a huge tummy and you ask “Sisi (sister) why didn’t you book” and she says that she was not aware that she was pregnant. How can you not be aware, nine months with something kicking every day, can you see that means this is unplanned pregnancy? That one is also a problem.”* (Midwife, MOU)

#### Frustration

Health infrastructure and system failures such as limited dedicated spaces for confidential counselling, shortages of equipment and drugs, and large patient to healthcare provider ratios are important deterrents for patients and causes of frustrations for both pregnant women and healthcare providers. Healthcare providers expressed their acute need for additional personnel, especially counsellors during night shifts, to cope with the many required tasks.

*You need to stay maybe +/- an hour with that one. And then there are complaints that you are delaying yet there is a lot to do. To counsel, take the blood, maybe they are clients who correlate with the new treatment, you know it is a lot.”* (Midwife, MOU)

Healthcare workers often must deal with system failures by juggling multiple responsibilities in addition to the already high volume of work, which will result in frustration.

*“Like if we can have somebody for the PCR* **(***Polymerase Chain Reaction test) thing (a test done for diagnosis of Human Immuno Deficiency Virus (HIV) in children). The PCR thing is a job, job, job…. It is a job plus in fact. Just because after delivering that mother, if that mother can have a complication, you have to deal with two things at the same time. You must go to the mother and make sure that the mother is stable and then arrange the treatment /transfer. If no transfer, then she must be stable before you came back to the baby where you can do the pricking of the heel, taking that blood. It is a process if you can see from a little baby you have to fill that five portions (dry blood spot card circles) with the blood. From there you must go down and complete the laboratory requisition form. Meanwhile, here you have also your maternity issues that you have to work with. We do all this by ourselves. So, if we have somebody to do the PCR thing and we deal with mother and the baby the normal way but this one (sample for PCR) somebody else is allocated to that one.”* (Midwife, MOU)

#### Empathy

Healthcare providers demonstrated empathy for pregnant women and described efforts expanded to educate women on the importance of ANC. They went to great lengths to overcome language and structural barriers by conducting one on one counselling.

*“Some of them (pregnant women) lack knowledge. Some of them have this thing of saying if I can just get the card to deliver. They do not care whether they attend only one visit or whatever it is. So, we are trying to educate them to know what we do to them when they come every time to the clinic. It’s not a matter of just getting a card so that the sister (nurse) would not say that you are not booked. It’s important to monitor the baby’s growth, blood results, blood pressures and whatever.”* (Registered nurse, PHC)

The quotes below highlight providers’ empathy for pregnant women and describe efforts to overcome the language and infrastructure barrier when they do counselling.

*“…We only have a problem with those who come from outside (other countries) because there is a language barrier. They have a problem with understanding Zulu even if you write a ‘love letter’ we don’t understand each other. If I see that the person cannot understand me if the person understands a little of English, I write down in English and the women can write in their language and I learn their language, I translate to other women.* (Midwife, MOU)

*“We are just squeezed ourselves here as you can see, and this does not allow privacy. We really need privacy, like if I have a pregnant woman who is HIV positive, I cannot talk to her in front of those others, I have to take her to the labour ward. What if in the labour ward there are deliveries that side, so there is no place I can go with that one for privacy?”* (Midwife, MOU)

## Discussion

In this paper, we examined the insight of healthcare providers into women’s reasons for starting ANC later than the South African National Department of Health recommended 20 weeks gestation, the impact of late ANC presentation on overall healthcare providers’ work experiences and healthcare providers’ response in their interactions with patients. Healthcare providers’ perspectives on the reasons why women present late for ANC reflected what they observed during their interactions with women. Our results suggest that healthcare providers are keenly aware of the reasons why women present late for ANC. Their observations on a day to day basis, lead to frustration, disbelief or empathy which in turn influences the way they treat these pregnant women. The results demonstrate that healthcare workers are aware of the need to educate women on the importance of ANC but sometimes their frustrations deter women from attending ANC early.

From the healthcare workers’ perspective, the study shows that pregnancy symptoms only are not enough for women to acknowledge that they are pregnant. Cultural beliefs about pregnancy viability play an important role in acknowledging that one is “officially” pregnant and seeking care [3]. Healthcare providers appear to despair that late presentation for ANC has become an immutable cultural norm and women replace ANC care with traditional care. Many studies have shown a widespread use of traditional medicine during pregnancy in South Africa and other African countries [26–28]. However, women’s need to be made aware of their health risks may not be satisfied when healthcare providers are fatalistic about the outcomes of health education efforts during ANC [29].

Our results show that even older women or women of higher gravida who have utilized ANC services before present late for ANC. We expect previously pregnant women to know the importance of early attendance of ANC considering that they were taught during counselling sessions in previous pregnancy ANC, but due to many reasons like cultural beliefs, travel costs and overcrowding at clinics, older women and women of higher gravida still present late for ANC. Healthcare providers may either overlook the need for repeat counselling among previously pregnant women or become more forceful in the counselling, for example by shouting as has been reported before [30]. Some higher gravida older women were perceived to delay ANC because of presumed knowledge and experience about pregnancy [31, 32].

Women with a planned pregnancy are generally more likely to book early for ANC than their counterparts with an unplanned pregnancy [6, 17, 33]. Additionally, the cost of attending a clinic visit, including travel cost, and overcrowding at clinics are known to reduce early bookings for ANC throughout Africa [17, 34–36].

Although healthcare providers do not have direct control over PHC personnel shortages, and cost and work-related patient-barriers to ANC, their insight into women’s motivations for seeking ANC can inform their efforts to educate their patients and adapt their own attitudes and responses to patient behaviours. Healthcare providers’ sensitivity to and attitude towards women’s health concerns are important determinants of patient demand for ANC services in the first pregnancy trimester [37]. South Africa has embarked on the Ideal Clinic program to guide healthcare providers in quality improvement processes by ensuring that an ideal clinic has good infrastructure, adequate staff, adequate medicine and supplies, good administrative processes and harnesses partner and stakeholder support. The impact of this intervention on healthcare providers’ awareness of elements of quality care and their capacity to address clinic process challenges is yet to be adequately evaluated. There is a need to implement the Ideal Clinic programme described above to address challenges experienced and expressed by PHC and MOU-based healthcare providers. Such a programme will undoubtedly influence their awareness, sensitivity and empathic attitude towards patient experiences in PHCs and support efforts to motivate healthcare providers to provide quality care.

### Limitations of the study

Our sample may not reflect the views of nurses and midwives more broadly in South Africa, but rather local opinions. However, the goal of this qualitative study was not to generalise our findings but rather to provide a rich, contextualised understanding of the reasons why women present late for ANC from healthcare providers’ perspective in Gauteng province, South Africa.

## Conclusion

The timing of ANC is influenced by the complex decisions women make during pregnancy, starting from accepting the pregnancy itself to acknowledging the need for antenatal care. To positively influence this decision making for the benefit of early ANC there is need to explore South African women’s perceptions of pregnancy and acknowledge that women’s perceptions of pregnancy and ANC differ from the Western views of care. Moreover, awareness programmes about the significance of early attendance of ANC is needed at the individual and community level, with a strong focus on the importance of early HIV treatment for PMTCT. In addition to factors such as personal barriers, system and service failures, negative attitudes and behaviours are major deterrents to care-seeking behaviour of pregnant women. Healthcare providers need to be encouraged and empowered to make necessary changes in patient flow challenges. More investigation is needed to better understand the patient-providers’ relationship in ANC services and ways of promoting positive interactions between these two parties to improve early and frequent attendance of ANC services in pregnancy.

## Data Availability

Qualitative data extracts are presented in the article to support the findings. The original transcripts are not available to the public as they may contain information that could compromise the confidentiality and anonymity of the participants.

## Abbreviations

ANC: Antenatal care
ART: Antiretroviral Treatment
CHC: Community Health Centres
MOU: Maternal Obstetric Units
PCR: Polymerase Chain Reaction
PHC: Primary Health Care
PMTCT: Prevention of Mother to Child Transmission
